# Hotspots of Sequence Variability in Gut Microbial Genes Encoding Pro-Inflammatory Factors Revealed by Oligotyping

**DOI:** 10.3389/fgene.2019.00631

**Published:** 2019-07-09

**Authors:** Ramón Gómez-Moreno, Rachell Martínez-Ramírez, Abiel Roche-Lima, Kelvin Carrasquillo-Carrión, Josué Pérez-Santiago, Abel Baerga-Ortiz

**Affiliations:** ^1^University of Puerto Rico Medical Sciences Campus, San Juan, Puerto Rico; ^2^Molecular Sciences Research Center, San Juan, Puerto Rico; ^3^CCRHD–RCMI Program University of Puerto Rico Medical Sciences Campus, San Juan, Puerto Rico; ^4^University of Puerto Rico Comprehensive Cancer Center, San Juan, Puerto Rico

**Keywords:** oligotyping, gut bacteria, colorectal neoplasia, colorectal cancer

## Abstract

The gut microbiota has been implicated in a number of normal and disease biological processes. Recent studies have identified a subset of gut bacterial genes as potentially involved in inflammatory processes. In this work, we explore the sequence variability for some of these bacterial genes using a combination of deep sequencing and *oligotyping*, a data analysis application that identifies mutational hotspots in short stretches of DNA. The genes for *pks island*, *tcpC* and *usp*, all harbored by certain strains of *E. coli* and all implicated in inflammation, were amplified by PCR directly from stool samples and subjected to deep amplicon sequencing. For comparison, the same genes were amplified from individual bacterial clones. The amplicons for *pks island* and *tcpC* from stool samples showed minimal levels of heterogeneity comparable with the individual clones. The amplicons for *usp* from stool samples, by contrast, revealed the presence of five distinct oligotypes in two different regions. Of these, the oligotype GT was found to be present in the control uropathogenic clinical isolate and also detected in stool samples from individuals with colorectal cancer (CRC). Mutational hotspots were mapped onto the USP protein, revealing possible substitutions around Leu110, Glu114, and Arg115 in the middle of the pyocin domain (Gln110, Gln114, and Thr115 in most healthy samples), and also Arg218 in the middle of the nuclease domain (His218 in the uropathogenic strain). All of these results suggest that a level of variability within bacterial pro-inflammatory genes could explain differences in bacterial virulence and phenotype.

## Introduction

The gut is a rich microbial ecosystem that contains numerous species, many of them implicated in human diseases. Direct relationships have been delineated between the presence of certain microbial species and inflammatory diseases such as Crohn’s disease, ulcerative colitis, and colorectal cancer (CRC) (Kostic et al., 2011; Martinez-Medina and Garcia-Gil, 2014; Brennan and Garrett, 2016). For instance, there are reports of associations between the presence of *Fusobacterium nucleatum, Akkermansia muciniphila*, enterotoxigenic* Bacteroides fragilis*, and *Streptococcus gallolyticus*, and CRC (Sears, 2009; Boleij et al., 2011; Kostic et al., 2011; Weir et al., 2013). More specifically, *Escherichia coli* has been found in the mucosa of CRC patients in higher numbers than in the mucosa of healthy individuals (Swidsinski et al., 1998; Martin et al., 2004).

It is increasingly clear that the involvement of *E. coli* in CRC takes place through mechanisms that are encoded by specific sets of genes (Bonnet et al., 2014; Raisch et al., 2014). These genes encoding genotoxic cyclomodulins and other pro-inflammatory molecules have been identified in certain strains of gram-negative bacteria. For instance, the *pks island* genes, a cluster of genes encoding enzymes for the production of the elusive natural product colibactin, has been found more frequently in mucosa samples from CRC individuals than in healthy donors (Arthur et al., 2012; Gomez-Moreno et al., 2019). Bacteria harboring the *pks* genes have also been found to induce the formation of tumors in AOX-treated mice (Nougayrede et al., 2006; Arthur et al., 2012). Similarly, the *tcpC* gene encoding a toll-like receptor antagonist that promotes the formation of kidney abscesses in mice has been found in strains of uropathogenic *E. coli* (Yadav et al., 2010). Also, the gene for the uropathogenic specific protein (*usp*) initially identified in *E. coli* from urinary tract infections encodes a DNAse with genotoxic activity that promotes cell death (Nipic et al., 2013).

Our group had previously established a protocol for the detection of specific pro-inflammatory genes in stool samples and established some preliminary associations with CRC (Gomez-Moreno et al., 2019). In that work, the presence of *pks island* genes and of *tcpC* was more common in stool samples from CRC patients and adenoma cases than in healthy controls. Also, the presence of the *usp* gene (encoding the uropathogenic specific protein) was found to be associated with colorectal neoplasia (Gomez-Moreno et al., 2019). Interestingly, a systematic search through the shotgun metagenomic databases also found the *usp* gene more frequently in samples or datasets from CRC patients than in the healthy population, suggesting its presence as a possible marker for CRC risk (Roche-Lima and Baerga-Ortiz, unpublished data).

While many of these genes seem to be present predominantly in CRC cases, they are not exclusive to CRC cases. Numerous samples from healthy individuals were found to harbor one or more of these pro-inflammatory genes. One possibility is that there are DNA sequence variants within the population and that some variants are more highly associated with disease or represent bacterial lineages more highly associated with disease.

Oligotyping is a method for the analysis of closely related sequences that is used to identify specific sites of sequence variability in relatively short PCR-amplified fragments. It relies on the parallel sequencing and subsequent comparison of millions of reads per sample to identify nucleotide positions with a phylogenetically relevant signal above the basal variability expected for a large set of information (Eren et al., 2016). The use of this entropy-based decomposition makes it possible to resolve closely related variants that differ by as little as one nucleotide at the amplified region (Eren et al., 2016). Oligotyping has been used to investigate ecological questions in diverse environments including the oral microbiota, gut microbiota, raw sewage, and soil (Mark Welch et al., 2014; Fisher et al., 2015; Turlapati et al., 2015; Vineis et al., 2016).

In an effort to identify sequence variants more associated with disease, we performed oligotyping analysis on DNA fragments amplified from the regions encoding selected pro-inflammatory genes that were found predominantly in CRC cases, but also in healthy controls (Gomez-Moreno et al., 2019).

## Methods

*Sample selection*. Stool samples had been provided by the Early Detection Research Network (EDRN) GLNE Clinical Validation Center to the UPR Stool Sample Repository (IRB Protocol A9560115) for a previous study (Gomez-Moreno et al., 2019). All samples were de-identified and labeled only with a numeric code. The samples chosen for this study were previously found to contain the *pks island*, *tcpC*, and the *usp* genes as reported by our group (Gomez-Moreno et al., 2019). **Table 1** summarizes the samples used and links them to the original numeric identifier given by the EDRN repository. The control strain IHE3034 was a generous donation from Dr. Eric Oswald from the University of Toulouse, and the EC640 strain, collected under protocol during a microbiological surveillance protocol, was a generous donation from Dr. Iraida E. Robledo from the University of Puerto Rico Medical Sciences Campus. The stool samples were thawed, and the bacterial DNA was extracted using the QIAgen Stool DNA Kit as described previously (Gómez-Moreno et al., 2014).

**Table 1:** Sample selection. Samples that had been previously been found to contain the genes for *pks island*, *tcpC*, and *usp* were selected for oligotyping analysis (Gomez-Moreno et al., 2019). All of the samples generated amplicons that correspond to specific regions of each gene, and those amplicons were subjected to deep sequencing. The number for each sample corresponds to the number from the EDRN (Early Detection Research Network) repository.

Bacterial gene	pks island	TcpC	usp
Healthy samples	3311681 3291671	3311681 3291671	3311681 3061661 3291671
Adenoma samples	3225691 3105661 2843671 3035661 3163691	3105661	3225691 3105661 2889661 3163691
CRC samples	3033671 3289651 3281671 3135661	3033671 3289651 3281671 3135661	2959661 3081661 3033671 3281671 3267661 3135661

*DNA amplification*. Polymerase chain reaction (PCR) was performed with sequence specific primers that also contained the overhang adapter sequence for Illumina (**Table 2**). The first-stage PCR was carried out by heating to 94°C for an initial denaturation step of 1 min, followed by 30 cycles of 94°C for 30 s, 30 s of the corresponding annealing temperature (**Table 2**), and a 3-min extension at 68°C, and finished with a 10-min extension step at 72°C. The second-stage PCR reaction was performed to add the Illumina index sequences as described in the Illumina protocol (Illumina Corp. Part# 15044223Rev.B)

**Table 2:** Primers and conditions for the PCR amplification of specific DNA sequences. Each oligonucleotide was synthesized with an additional DNA sequence marked in bold for the purpose of indexing.

Gene	Primer nucleotide sequence	Annealing T (°C)	Product size (bp)
pks island	Forward: **TCGTCGGCAGCGTCAGATGTGTATAAGAGACAG** GTTTTGCTCGCCAGATAGTCATTC	63	800
	Reverse: **GTCTCGTGGGCTCGGAGATGTGTATAAGAGACAG** CAGTTCGGGTATGTGTGGAAGG		
tcpC	Forward: **TCGTCGGCAGCGTCAGATGTGTATAAGAGACAG** TCGGCGATAGCTTAAGGAGA	63	283
	Reverse: **GTCTCGTGGGCTCGGAGATGTGTATAAGAGACAG** CCGCCAAATAATGGCTGTAT		
usp	Forward: **TCGTCGGCAGCGTCAGATGTGTATAAGAGACAG** GGTGTTCATACGGGTGAAGG	63	685
	Reverse: **GTCTCGTGGGCTCGGAGATGTGTATAAGAGACA** GCTCAGGGACATAGGGGGAAT		

*Quality assessment*. First-stage PCR products were visualized by agarose gel electrophoresis (1%) stained with GelRed^™^ (Biotium), followed by gel extraction using the QIAquick Gel Extraction kit. The concentration and purity of each amplicon was determined using the nanodrop 2000c (Thermo Scientific). Second-stage PCR quality was tested using the Agilent 2100 Bioanalyzer, and the concentrations were determined using Qubit 2.0 fluorometer (Invitrogen).

*Illumina sequencing*. DNA sequencing was carried out by paired end sequencing using the MiSeq Sequencing Platform (Illumina, San Diego, CA). All quality controls and pre-processing were performed using the fastx-tool kit (http://hannonlab.cshl.edu/fastx_toolkit/). Reads with a quality score of 30 on at least 80% of the bases were kept for further analysis. For each gene of interest, we combined all samples into a single FASTA file and formatted as required for running the oligotyping pipeline. We added gaps to the shorter reads to match with the longest reads as all reads should have the same length and then performed entropy analysis.

All of the processed sequence data that was used as input for Oligotyping can be found in the NCBI Bioproject database under Accession PRJNA551701.

*Oligotyping*. We performed oligotyping analysis using the pipeline version 2.1 (available from http://merenlab.org/software/oligotyping/) (Eren et al., 2013). After the initial calculation of Shannon entropy using the analyze-entropy script in the oligotyping pipeline, we ran the method separately for the R1 and R2 reads so as to identify the nucleotide positions with the highest sequence variability. Using the R1 reads generated from the *usp* amplicon as an example, oligotyping analysis was performed on 5,855,441 reads pooled together using three components automatically selected form the highest entropy values following the initial entropy analysis. To reduce the noise, each oligotype required to have a most abundant unique sequence with a minimum abundance of 10. Oligotypes that did not meet this criterion were removed from the analysis. For the R2 reads, the analysis was performed on 4,412,715 reads as described for R1 reads. The most abundant oligotypes in each sample were quantified and plotted. As a control, all the datasets contained the amplicons generated from a bacterial isolate, which was supposed to show no sequence variation whatsoever.

*Searching for usp oligotypes in published metagenomic datasets*. We analyzed metagenomic DNA sequence data obtained from the European Nucleotide Archive (https://www.ebi.ac.uk/ena) database (accession no. PRJEB12449). These shotgun gut microbiome datasets were obtained from a case–control study conducted with stool samples collected at two locations: Washington DC and France (Vogtmann et al., 2016). We had already determined that the sequences for *pks island*, *tcpC*, and *usp* were present in these datasets by performing a BLASTN search on the downloaded FASTQ.gz files (Roche-Lima et al., unpublished results). However, the frequency of hits in the Washington DC datasets was very low (in some cases zero). Thus, we decided to carry out the sequence analysis using the datasets from the France population. We used the same BLASTN method to search for the *usp*-positive reads using the following queries: GAACATGCAGTGGAACGAGCAGACGCAGCG and ACAGGGCGCGGCGTCCCGCTT. These searches were intended as a validation of the sites of sequence variation for this gene locus.

*Three-dimensional protein models*. A three-dimensional model for USP was built using the I-TASSER (Iterative Threading ASSEmbly Refinement) server and visualized using VMD (Humphrey et al., 1996; Yang et al., 2015). The I-Tasser server selected the crystal structure of the colicin D central domain from *Escherichia coli* (PDB ID: 5ZNM) as the best template for USP.

## Results

For the *pks island* genes, a fragment of 800 bp from the *clbN* gene was amplified directly from 11 stool samples that had been found to be positive for this gene (2 healthy, 5 adenomas, and 4 CRC). Similarly for the *tcpC* gene, a fragment of 283 bp was amplified from seven stool samples (two healthy, one adenoma, and four CRC). Finally, for the *usp* gene, a fragment of 685 bp was amplified from 13 samples (3 healthy, 4 adenomas, and 6 CRC). As a control, we PCR-amplified the same set of genes using the DNA from single bacterial isolate, IHE3034, which is *pks* ^+^, and EC640, which is *tcpC* ^+^ and *usp* ^+^, which was initially obtained from the urine of a 5-month-old patient with a urinary tract infection at the University of Puerto Rico Pediatric Hospital and was found to be positive for all three genes (Wolter et al., 2009).

**Figure 1:**
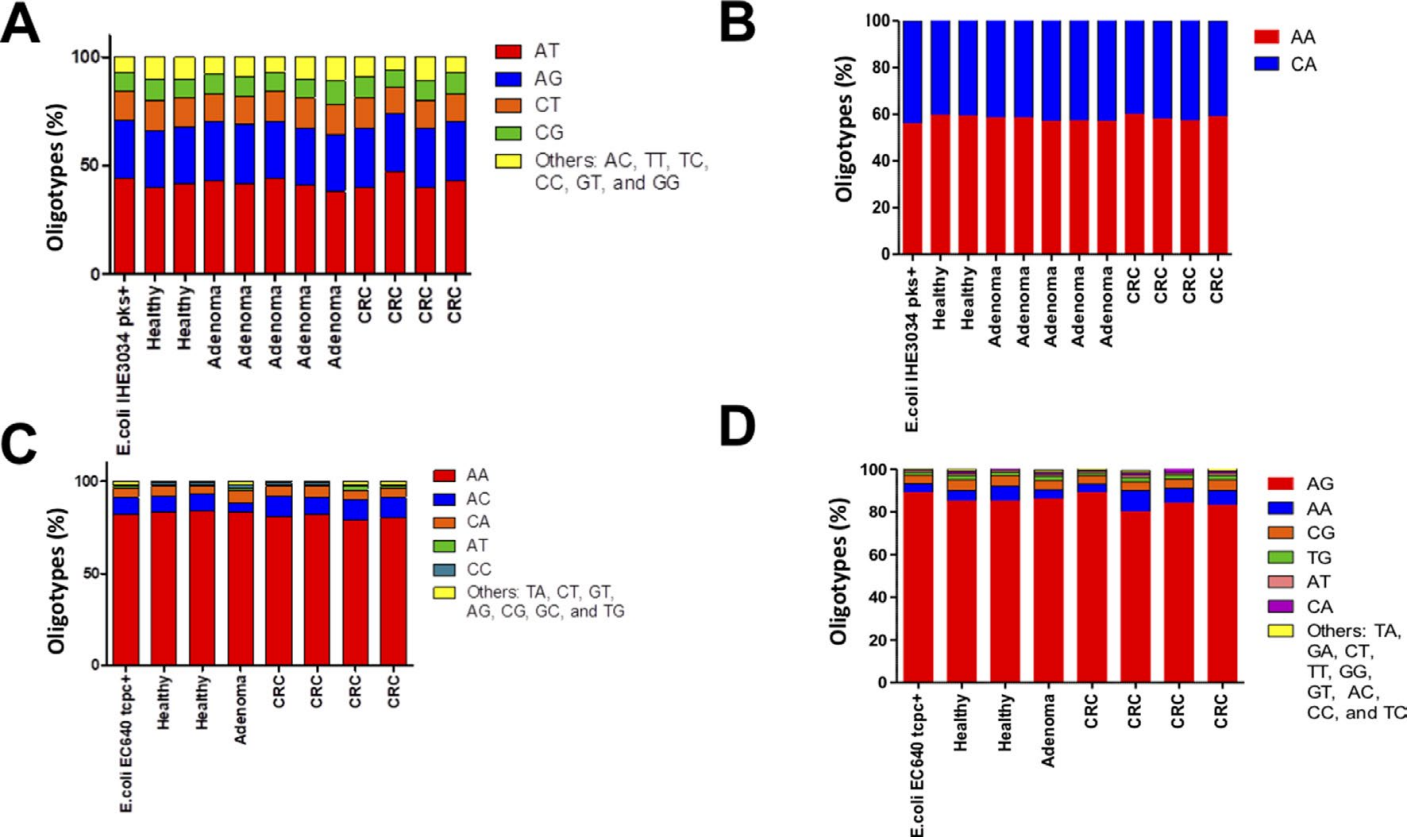
Distribution of oligotypes for the DNA fragments amplified from stool samples. The genes corresponding to **(A)** *pks island* R1 **(B)**,* pks island* R2 **(C)**, *tcpC* R1, and **(D)** *tcpC* R2 were analyzed by oligotyping [Eren et al., 2013]. The distribution of oligotypes amplified from stool samples is similar to the distribution resulting from a single clone for strain IHE3034 (pks^+^) and strain EC640 (tcpC^+^), suggesting low sequence variability for these tow genes in the gut microbiome.

**Figure 2:**
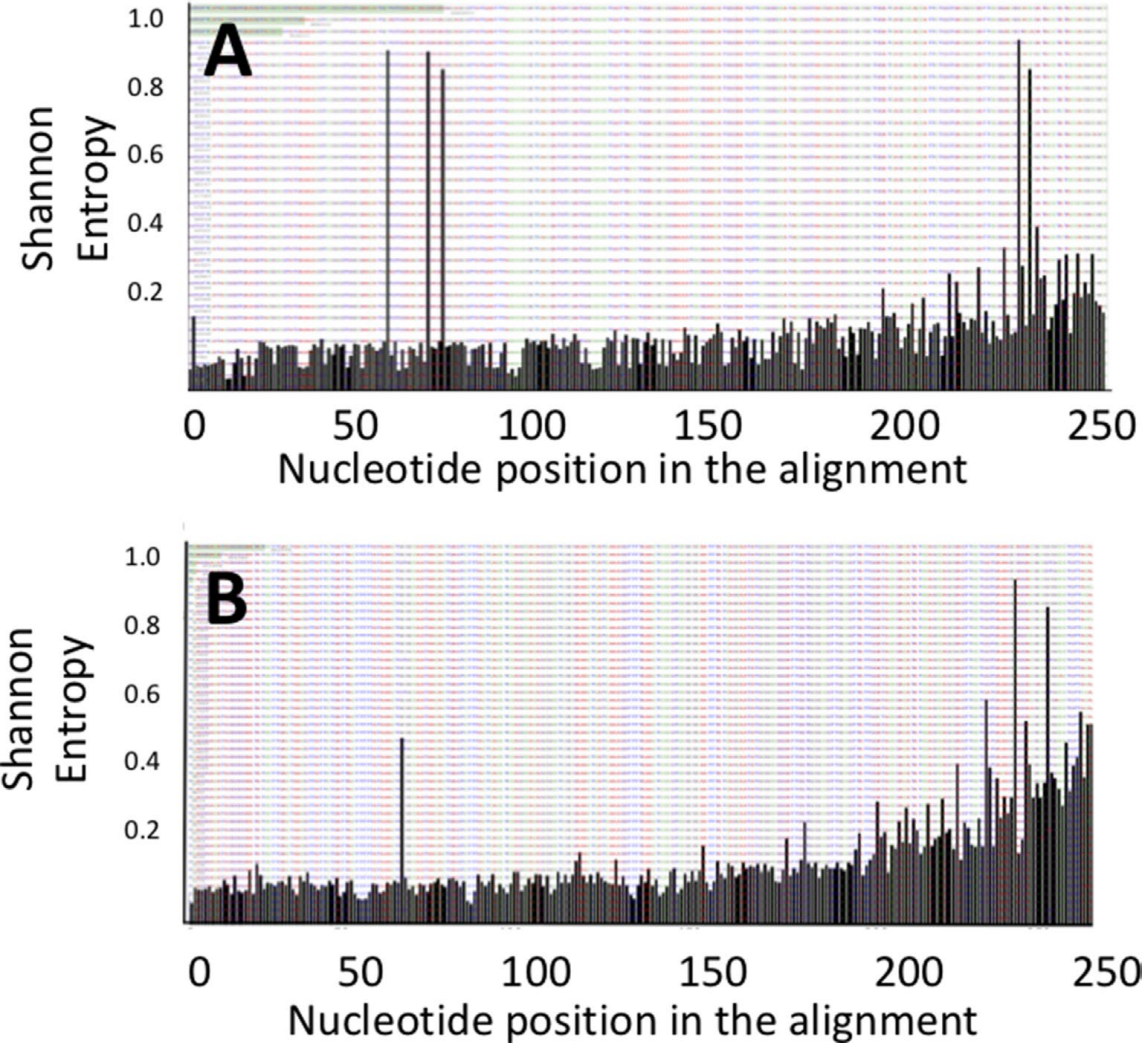
Shannon entropy plots for the *usp* sequences amplified from stool samples reveal positions of nucleotide variability for the **(A)** upstream R1 and **(B)** downstream R2 portions of the *usp* amplicon.

The DNA fragments corresponding to the genes *pks* and *tcpC* that were amplified from patient-derived stool samples showed a similar oligotype profile as the control isolate strain (**Figure 1**, the control is the leftmost bar in all panels); a level of sequence heterogeneity consistent with a single population and possibly resulting from method-related phenomena as will be addressed in the discussion section. By contrast, the DNA fragments amplified from stool samples containing *usp* showed clear hotspots of sequence variation (**Figure 2**). In the R1 direction, three sites of variability were identified: positions 329, 340, and 344 of the gene (**Figure 2A**). In the R2 direction, two sites of variation were identified: positions 653 and 662 of the gene (**Figure 2B**).

**Figure 3:**
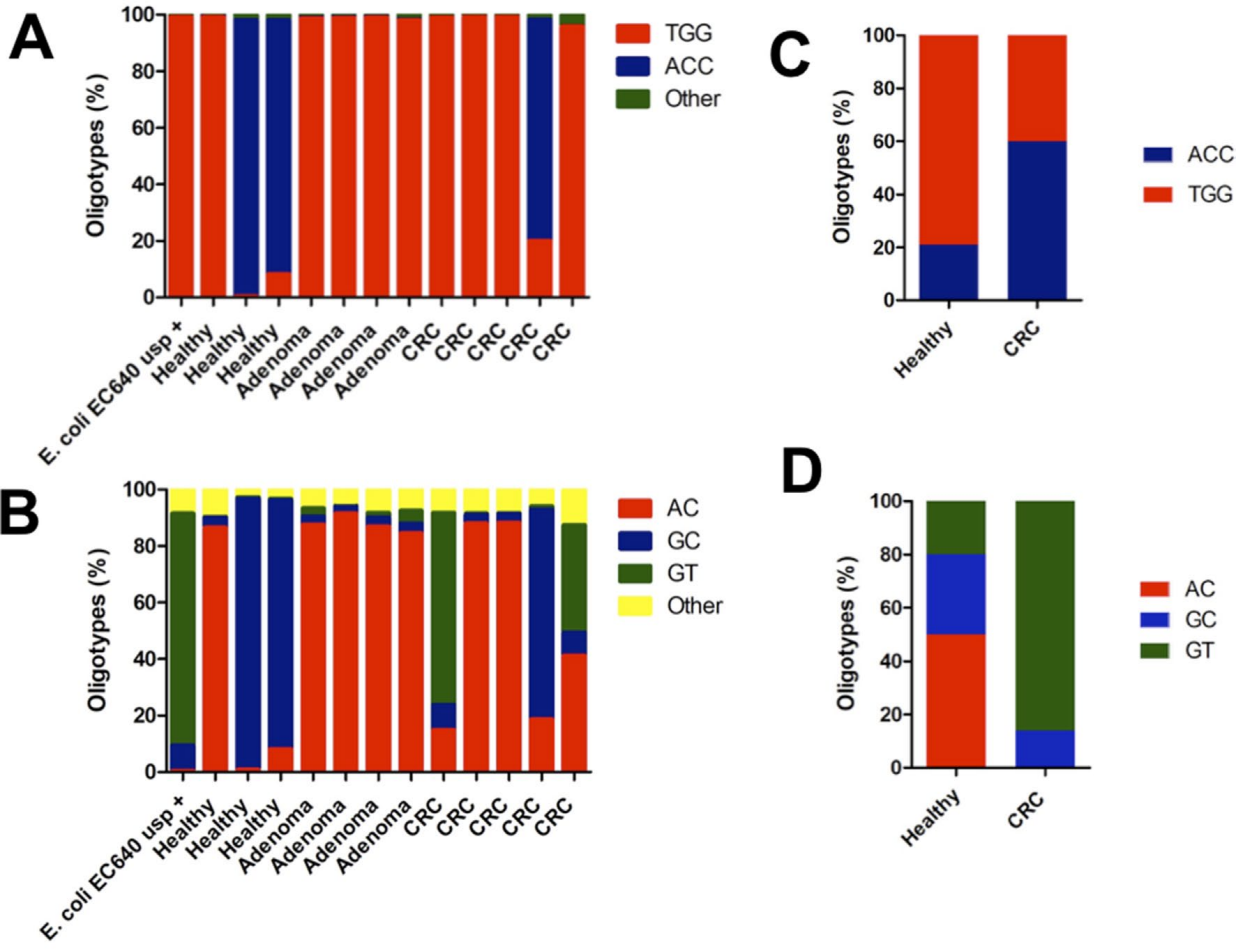
The distribution of oligotypes for the DNA fragments obtained from the amplification of the *usp* gene reveals patient-specific lineages when sequenced in the **(A) **forward direction R1 and in **(B)** reverse direction R2. The control EC640, a uropathogenic strain, is mostly the TGG oligotype in the forward direction and the GT oligotype in the reverse direction. The two patients who harbor this GT oligotype both have CRC, and the GT oligotype is also found more frequently in uropathogenic strains. Oligotyping results compared the data found in the metagenomic datasets corresponding to a CRC case–control study. **(C)** The same oligotypes, TGG and ACC, were found in the region covered in the R1 reads of the present study. **(D)** Also, the same oligotypes, AC, GC, and GT, were found in the DNA region covered by the R2 reads in the present study.

Sequence analysis revealed two main oligotypes present in different proportions in the R1 (**Figure 3A**) direction and three main oligotypes in the R2 direction (**Figure 3B**). The oligotype labeled “TGG” was the most abundant one with a thymine in position 329 and guanosines in positions 340 and 344 of the gene, respectively (**Figure 3A**). This oligotype was also the most abundant in the EC640 uropathogenic control strain and also present in most of the CRC and adenoma samples (**Figure 3A**). The other abundant oligotype “ACC” contained adenine in position 329 and cytosines in positions 340 and 344 and was present in most of the healthy samples (**Figure 3A**).

Since we performed paired-end sequencing, there was another set of data for the sequence at the opposite end of each of the amplicons. Again, the distribution of oligotypes for *pks* and *tcpC* reveals negligible heterogeneity compared to the control (**Figure 1C and D**). By contrast, the *usp* sequence reveals three main oligotypes, “AC” being the most abundant one and “GT” being the one associated with the uropathogenic control strain and with two CRC cases (**Figure 3B**).

In order to validate the presence of these hotspots of sequence variability along the *usp* gene, we analyzed the sequences from previously published shotgun metagenomic datasets for CRC individuals, compared with age- and gender-matched controls (Vogtmann et al., 2016). To our surprise, we observed in these published datasets the exact same sites of sequence variability that we report for our samples. Despite the fact that the samples were collected at different times and in different continents, they all contain a similar distribution of oligotypes. The sequence datasets from the French population showed no difference in the total number of hits for the *usp* gene between CRC and healthy controls (16 hits vs 15 hits; data not shown). In terms of oligotype distribution, the TGG oligotype was also found to be the most abundant, with the ACC oligotype marginally associated with disease (**Figure 3C**). In the other end of the gene, oligotype GT is also most associated with CRC (**Figure 3D**).

**Figure 4:**
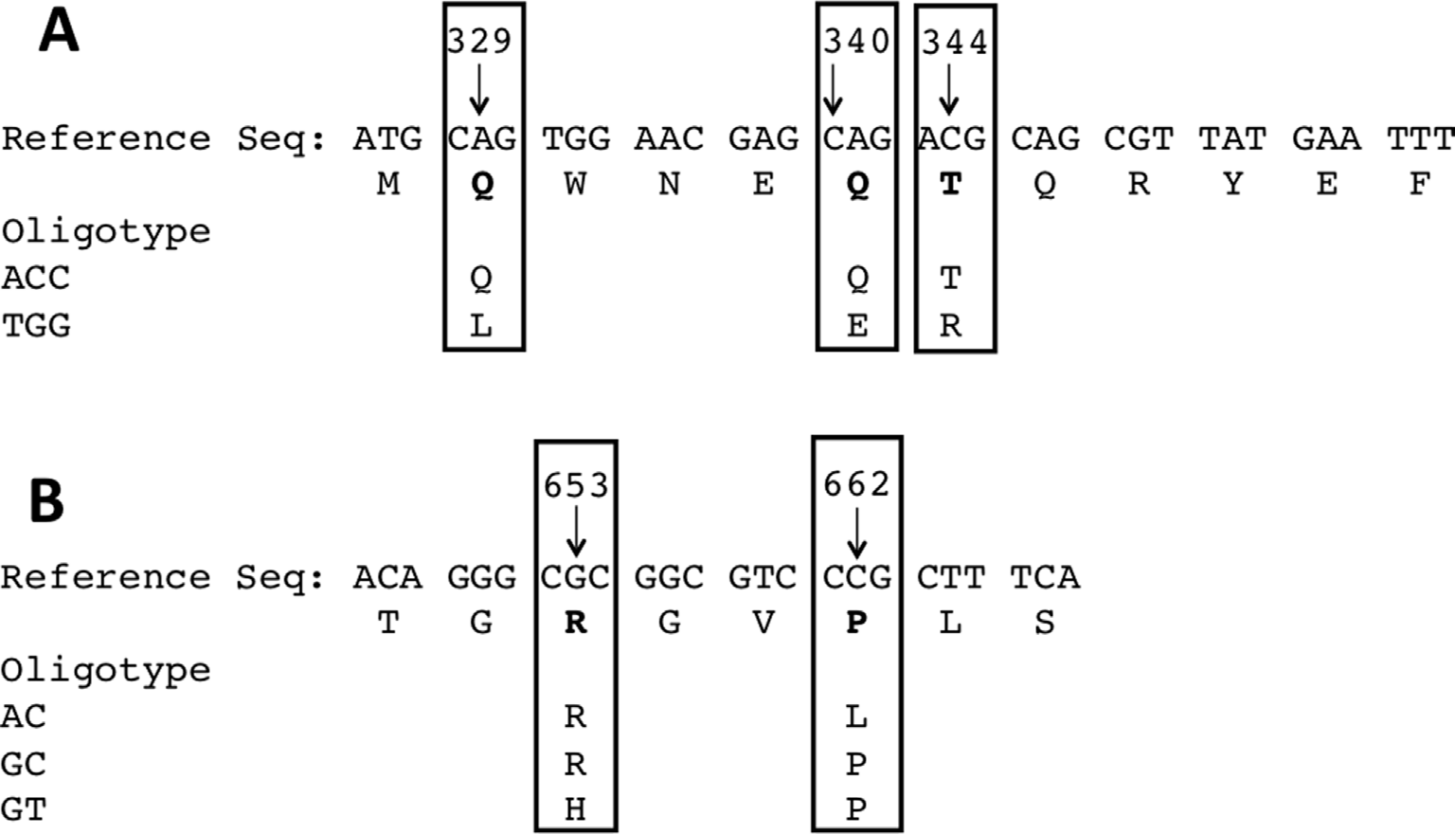
**(A)** Variability around positions 329, 340, and 344 result in amino acid substitutions along the protein sequence from Leu110/Glu114/Arg115 to Gln110/Gln114/Thr115. **(B)** Similarly, in the opposite end, the most abundant oligotype encodes Arg218/Leu221, whereas the oligotype most frequently seen in uropathogenic strains encodes His218/Pro221.

**Figure 5:**
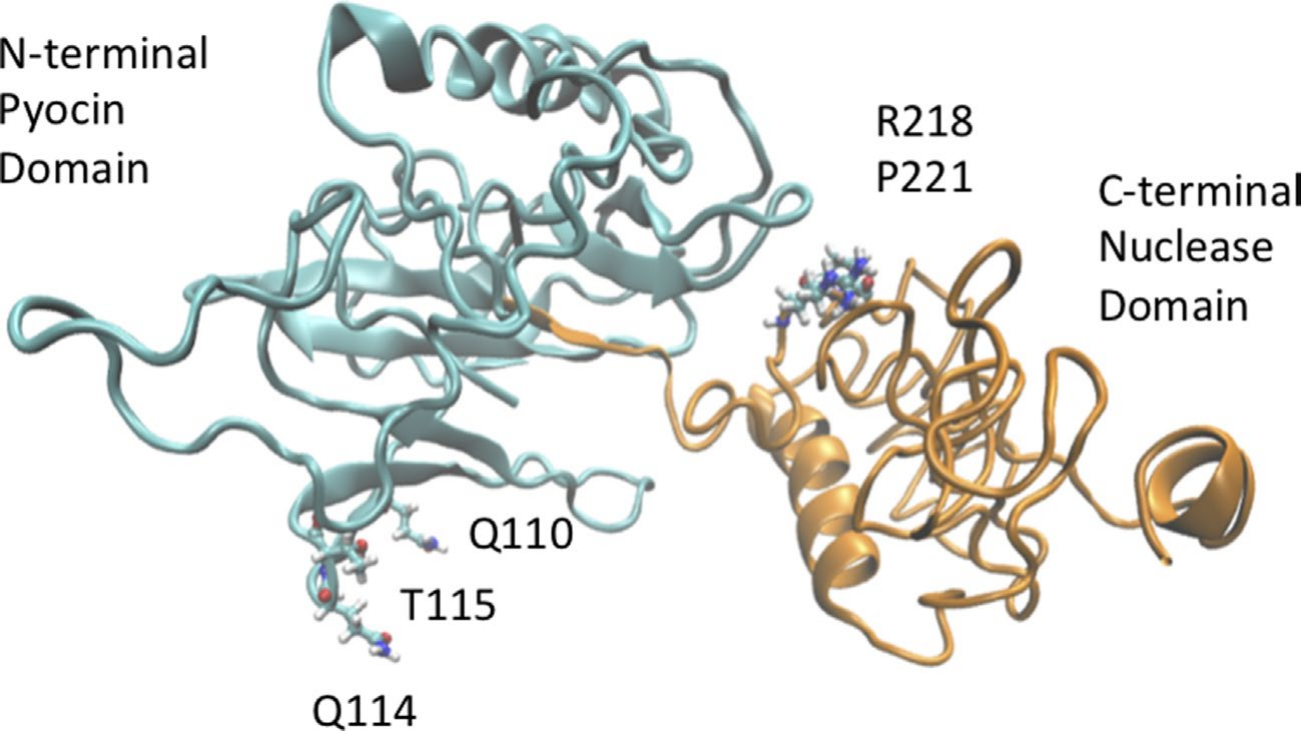
A three-dimensional model of USP was generated using i-TASSER (Eren et al., 2013) and visualized using VMD (Vogtmann et al., 2016) to show the location of possible sites of amino acid variation. Position 110 within the pyocin-like domain of USP (cyan domain) can be occupied either by a Leu or Gln residue. Similarly, positions 218 (Arg/His) and 221 (Leu/Pro) of the nuclease domain (green domain) are also hotspots of sequence variation.

The base pair substitutions in these oligotypes represent a change in amino acid sequences of the resulting USP protein variants, which could result in functional differences. For instance, the abundant TGG oligotype encodes Leu, Glu, and Arg in positions 110, 114, and 115 of the USP protein, whereas the ACC oligotype encodes Gln, Gln, and Thr in those same positions (**Figure 4**, R1). These amino acids are all located in the N-terminal pyocin-like domain, which is thought to be involved in toxin targeting (**Figure 5**). At the other end of the protein is the C-terminal DNAase domain, in which the abundant oligotype AC encodes for Arg and Leu in positions 218 and 221, respectively, whereas the CRC to His218 and Pro221 in the uropathogenic oligotype GT (**Figure 4**, R2). Although these substitutions in the C-terminal domain do not take place near the nuclease active site, they could affect activity through protein structural effects.

Although the notion that sequence variation can have an effect on USP activity or phenotype remains to be validated, it is clear that there is enough variability in the *usp* gene to define bacterial lineages that could be further correlated with disease risk or clinical outcome.

## Discussion

In previously published work, our group had reported the presence of several bacterial genes known to promote inflammation in stool samples from individuals with CRC (Gomez-Moreno et al., 2019). One of these genes was *usp*, which encodes a nuclease enzyme typically found in uropathogenic strains of *E. coli*. In this work, we explore the sequence variability for these genes in human stool and the possible association of specific sequence variants with disease. Interestingly, we found no significant variability in the sequences encoding *tcpC* or *pks island* genes, suggesting that at least in those regions that were amplified, only a single sequence or lineage exists within the population tested. By contrast, the *usp* gene had five main oligotypes, which could, if expressed, give rise to several USP enzyme variants. One of these variants, GT, was present in the clinical isolate that was used as a technical control, which was obtained from a patient with a urinary tract infection. Two samples contained this oligotype predominantly and both were CRC patients. Clearly, the establishment of a correlation between this particular lineage of *usp* and CRC will require a larger patient sample.

One limitation of the present study is the small number of stool samples that were analyzed, which makes statistical analysis very difficult. Since the original study was a pilot study, it involved a limited number of samples that had been carefully characterized in terms of demographics and diagnosis status (Gomez-Moreno et al., 2019). However, despite the limited number of samples in our study, our results are validated when they are compared with the published sequences in metagenomic (or shotgun) datasets (Vogtmann et al., 2016). The exact same sites of DNA sequence variation were found in the published datasets as were reported in this study. Also, there was a marginal association between the presence of one of the oligotypes, GT, and CRC, as observed in our experiments and confirmed in the published datasets (**Figure 3**). Our results are also consistent with earlier reports of *usp* sequences from isolated clinical strains in which they also report a number of sites of DNA sequence variation that coincide with the sites uncovered in this report (Nakano et al., 2001).

Another limitation of this report is that we only measure the sequence variability on a portion of the total sequence space for the genes under study. For instance, the *tcpC* gene is 924 base pairs long, but we are only amplifying a fragment of 283 base pairs, which was the only PCR product obtained from bacterial DNA purified directly from human stool as a template. We attempted different oligonucleotide combinations to yield longer PCR fragments, but those resulted in mixtures of unrelated DNA sequences that were difficult to deconvolute. Similarly, the *usp* gene is 1041 bases long, but we only were able to amplify a fragment that was 685 bases long and the coverage of the forward and reverse sequencing was roughly 300 bases on each end. Thus, it is possible that investigating the sequence variability of whole genes will require several amplification reactions to allow for the coverage of the entire gene sequence.

An unexpected finding was the sequence variability of DNA amplified from the individual bacterial clones that we used as controls. We expected these control strains to yield single oligotypes for each of the genes. However, a number of distinct oligotypes were detected for all of the fragments amplified from the control strains (**Figures 1** and **3**, leftmost bar). This apparently broad distribution of oligotypes in what should be a single clone could be due to a PCR incorporation error or to the formation of sequence chimeras (Haas et al., 2011; Schloss et al., 2011). Also, it cannot be ruled out that the sequence heterogeneity could stem from actual cellular diversification within a single colony, a phenomenon that has been observed as the presence of distinct phenotypes within a bacterial colony (Freeman et al., 1996; Nahku et al., 2011; Saint-Ruf et al., 2014).

Taken together, our results reveal the presence of sequence variants of the *usp* gene in patient samples, raising the possibility that some variants may have different activity and toxicity profiles.

## Data Availability

The data supporting the conclusions of this manuscript is available in the the NCBI Bioproject database under Accession PRJNA551701.

## Author Contributions

RG-M and AB-O designed the study. AB-O obtained the funding necessary for the study. RG-M performed the experiments. RG-M, RM-R, AR-L, KC-C, JP-S, and AB-O performed the data analyses and interpretation of the data. RM-R and AB-O prepared the final figures. All authors contributed in the preparation of tables and figures and in drafting the manuscript. Some of this work was published as part of the doctoral thesis of RG-M.

## Funding

This work was supported in part by the National Institutes of Health grant R25GM061838 (NIGMS-RISE Program) to RGM. Oligonucleotides were purchased from an institutional facility supported by NIH Grant G12RR03051 (RCMI Program). Sequencing was carried out at the SGF facility, which is funded through the PR-INBRE program (NIGMS P20 GM103475). The work was also supported by the Center for Collaborative Research in Health Disparities Award Number G12 MD007600 from the National Institute on Minority Health and Health Disparities.

## Conflicts of Interest Statement

The authors declare that the research was conducted in the absence of any commercial or financial relationships that could be construed as a potential conflict of interest.
